# Overexpression of the Interferon-Inducible Isoform 4 of *NCOA7* Dissects the Entry Route of Enveloped Viruses and Demonstrates that HIV Enters Cells via Fusion at the Plasma Membrane

**DOI:** 10.3390/v11020121

**Published:** 2019-01-29

**Authors:** Nikolas Herold

**Affiliations:** 1 Childhood Cancer Research Unit, Department of Women’s and Children’s Health, Karolinska Institutet, 171 76 Stockholm, Sweden; nikolas.herold@ki.se; 2 Paediatric Oncology, Theme Women’s and Children’s Health, Karolinska University Hospital, 171 76 Stockholm, Sweden

**Keywords:** human immunodeficiency virus, entry, endocytosis, fusion, NCOA7, VSV-G, Env, plasma membrane

## Abstract

The HIV-1 entry-route is a matter of ongoing controversy, and there is evidence for fusion either at the cell surface or from within endosomes. A recent report demonstrated that isoform 4 of nuclear receptor coactivator 7 (NCOA7_iso4_) interacts with endolysosomal vacuolar-type H^+^-ATPase (V-ATPase), increasing lytic activity and thereby severely affecting the entry of vesicular stomatitis virus glycoprotein (VSV-G)-mediated, but not HIV-Env-mediated, entry and infection. As basal expression of NCOA7_iso4_ is low in the absence of type-1 interferons, its overexpression is a novel tool to study viral entry.

Enveloped viruses need to fuse with a cellular membrane to deliver their viral content to the cytosol of their host cell. Following receptor engagement, fusion can occur directly at the plasma membrane or subsequent to endocytosis. For retroviruses, both modes of entry exist: ecotropic murine leukaemia virus and avian leukosis virus are paradigmatic examples for fusion at the plasma membrane and from within endosomes, respectively [1,2]. Cellular uptake of HIV-1 particles into endomembrane compartments has been identified previously [3,4,5], but has traditionally been considered a non-productive and/or recycling pathway [3,6]. Several lines of evidence have been reported for the plasma membrane being the primary site of HIV-1 entry: (i) the endocytic signal peptide in CD4 is dispensable for HIV-1 infection [7]; (ii) expression of HIV-Env efficiently mediates cell-to-cell fusion [8]; and (iii) HIV-1 infection is not dependent on a low-pH cue [4,9]. Nevertheless, direct visualisation of HIV-1 fusion at endomembranes has been reported [10]. Furthermore, at least in non-physiological adherent model cell lines, inhibitors of acidification led to enhanced HIV-1 infection, suggesting that HIV-1 fusion at endomembranes is increased if lysosomal degradation is prevented [11,12]. In induced pluripotent stem cell-derived macrophages that are endocytically more active as compared to T-cells, the forced increase of fusion at the plasma membrane by overexpression of Lck (reducing CD4 endocytosis) led to increased reverse transcription, but did not lead to increased productive infection [13]. In addition, the inhibition of dynamin-dependent endocytosis by the overexpression of a dominant-negative mutant as well as small molecule inhibitors of clathrin-mediated endocytosis reduced HIV-1 infectivity in adherent reporter cell lines [14,15], even though these approaches did not affect fusion and infection efficiency in T-cells [16]. However, dynamin-2 has been reported to contribute to fusion at the plasma membrane in T-cells by stabilising the fusion pore [17,18]. A similar role for dynamin-2 has been suggested for the cell-to-cell transmission of HIV-1; nevertheless, the site of fusion following cell-to-cell transmission of HIV-1 is as controversial as cell-free virus entry [19,20].

More recently, work by Miyauchi and co-workers suggested that HIV-1 enters T-cells exclusively via endocytosis [21,22]. These results are based on single-virus tracking that uses viral particles carrying separate labels in their lipid membrane and their viral core. Hence, live-cell imaging and the monitoring of the site, sequence and type of colour separation allowed the direct visualisation and quantification of putative fusion events. In conflict with these data, exploiting the fact that HIV-Env-mediated fusion is arrested at temperatures below 23 °C, while endocytosis still occurs, we showed by time-of-addition experiments with the membrane-impermeable fusion inhibitor T-20 that HIV-1 fusion and infection are not dependent on endocytosis in T-cells [16]. This was true for both primary-patient isolated and lab-adapted HIV-1 glycoproteins, irrespective of their co-receptor tropism [16]. The discrepancies between our work and the work by Miyauchi et al. have been discussed, but could not be completely resolved [23,24].

The family of interferon-induced transmembrane proteins (IFITMs) restrict virus–cell fusion, but cannot faithfully tell the mode of entry apart, as they inhibit fusion both at the plasma membrane and from within endosomes [25]. Nevertheless, one report inferred from the differential susceptibility of CXCR4- and CCR5-tropic HIV-1 envelope glycoproteins to distinct IFITM homologues that co-receptor tropism might define the subcellular site of fusion; IFITM-2 and 3, located at endosomes and lysosomes, restricted X4-Env, whilst IFITM-1, predominantly found at the plasma membrane, restricted R5-Env bearing HIV-1 [19,26].

In the December issue of *Nature Microbiology*, Doyle et al. demonstrate that another interferon-inducible factor, isoform 4 of nuclear receptor coactivator 7 (NCOA7_iso4_), inhibits fusion and infection triggered by viral glycoproteins known to mediate entry at endomembranes, but not of glycoproteins that fuse at the plasma membrane [27]. Mechanistically, this is mediated by direct NCOA7_iso4_–V-ATPase interaction at endosomes, leading to increased acidic proteolysis of the endosomal cargo. Given that basal NCOA7_iso4_ expression is low in the absence of interferon, a major implication of this study is that sensitivity to overexpression of NCOA7_iso4_ can dissect whether an enveloped virus enters cells via fusion at the plasma membrane or from within endosomes, and will certainly become a standard assay for studies into early viral life-cycles. Accordingly, VSV-G-pseudotyping sensitises HIV-1 to NCOA7_iso4_ restriction, whereas wild-type HIV-1 is resistant to NCOA7_iso4_.

Effectively, this study shows that the entry of HIV-1 does not require endocytosis and thus occurs *bona fide* at the plasma membrane, at least under experimental conditions using the glioma cell line U87MG CD4^+^ CXCR4^+^ and X4-tropic lab-adapted NL4-3-Env. Future work will have to address whether the overexpression of NCOA7_iso4_ also ablates HIV-1 infection in primary target cells of HIV-1, namely T-cells and macrophages. Furthermore, since co-receptor usage has been implicated in the viral entry route [26] and lab adaptation might alter entry requirements, primary Env isolates should be subjected to this novel assay. This promises to contribute importantly to, or even resolve, the controversy of the HIV-1 entry route.
